# A Multifactorial Approach for Sarcopenia Assessment: A Literature Review

**DOI:** 10.3390/biology10121354

**Published:** 2021-12-20

**Authors:** Rashmi Supriya, Kumar Purnendu Singh, Yang Gao, Feifei Li, Frédéric Dutheil, Julien S. Baker

**Affiliations:** 1 Centre for Health and Exercise Science Research, Sarcopenic Research Unit, Department of Sport, Physical Education and Health, Hong Kong Baptist University, Kowloon Tong, Hong Kong 999077, China; gaoyang@hkbu.edu.hk (Y.G.); lifeifei@hkbu.edu.hk (F.L.); jsbaker@hkbu.edu.hk (J.S.B.); 2 FEBT, School of Environment, Resources and Development, Asian Institute of Technology, Klong Luang, Bangkok 12120, Thailand; purnendusin@gmail.com; 3 University Clermont Auvergne, CNRS, LaPSCo, Physiological and Psychosocial Stress, CHU Clermont-Ferrand, University Hospital of Clermont-Ferrand, Preventive and Occupational Medicine, Witty Fit, F-63000 Clermont-Ferrand, France; frederic.dutheil@uca.fr

**Keywords:** sarcopenia, biomarkers, genetic factors, psychosocial factors, behavioral risk factors, literature review

## Abstract

**Simple Summary:**

Sarcopenia is characterized by an accelerated decline in skeletal muscle mass and strength, which results in poor quality of life, disability, and death. In the literature, sarcopenia is defined as the progressive breakdown of muscle tissue. The prevalence ranges from 5% to 13% in people 60–70 years old and from 11% to 50% in people older than 80 years. The comparison of risk factors associated with sarcopenia based on the European Working Group on Sarcopenia (1 and 2) in Older People, the Asian Working Group for Sarcopenia (1 and 2), the International Working Group on Sarcopenia, and the Foundation for the National Institutes of Health revealed no consistent patterns. Accordingly, the identification of a single risk factor for sarcopenia is unpredictable. Due to its “multifactorial” pathogenesis related to the involvement of a multitude of factors. In this review, we summarize 13 relevant risk factors associated with this disease that are important to consider prior to embarking on any related sarcopenia research. We suggest that researchers should concentrate on the biology of sarcopenia to develop a uniform consensus for screening this condition. In this review, we identify 50 biochemical markers across six pathways that have previously been investigated in subjects with sarcopenia. We suggest that these summarized biomarkers can be considered in future diagnosis to determine the biology of this disorder, thereby contributing to further research findings. As a result, a uniform consensus may also need to be established for screening and defining the disease. Sarcopenia is associated with a number of adverse economic and social outcomes, including disability, hospitalization, and death. In relation to this, we propose that we need to develop strategies including exercise interventions in the COVID-19 era to delay the onset and effects of sarcopenia. This suggestion should impact on sarcopenia’s primary and secondary outcomes, including physical, medical, social, and financial interactions.

**Abstract:**

Sarcopenia refers to a progressive and generalized weakness of skeletal muscle as individuals age. Sarcopenia usually occurs after the age of 60 years and is associated with a persistent decline in muscle strength, function, and quality. A comparison of the risk factors associated with sarcopenia based on the European Working Group on Sarcopenia (1 and 2) in Older People, the Asian Working Group for Sarcopenia (1 and 2), the International Working Group on Sarcopenia, and the Foundation for the National Institutes of Health revealed no consistent patterns. Accordingly, the identification of a single risk factor for sarcopenia is unpredictable due to its “multifactorial” pathogenesis, with the involvement of a multitude of factors. Therefore, the first aim of this review was to outline and propose that the multiple factors associated with sarcopenia need to be considered in combination in the design of new experimentation in this area. A secondary aim was to highlight the biochemical risk factors that are already identified in subjects with sarcopenia to assist scientists in understanding the biology of the pathophysiological mechanisms affecting the old people with sarcopenia. We also briefly discuss primary outcomes (physical) and secondary outcomes (social and financial) of sarcopenia. For future investigative purposes, this comprehensive review may be useful in considering important risk factors in the utilization of a panel of biomarkers emanating from all pathways involved in the pathogenesis of this disease. This may help to establish a uniform consensus for screening and defining this disease. Considering the COVID-19 pandemic, its impact may be exacerbated in older populations, which requires immediate attention. Here, we briefly suggest strategies for advancing the development of smart technologies to deliver exercise in the COVID-19 era in an attempt regress the onset of sarcopenia. These strategies may also have an impact on sarcopenia’s primary and secondary outcomes.

## 1. Introduction

Sarcopenia is a clinical condition in which skeletal muscle mass and strength gradually decline, leading to adverse outcomes including poor quality of life, disability, and death [1,2]. Sarcopenia is defined in the literature as a progressive degeneration of muscle tissue, with a prevalence range of 5–13% in people 60–70 years of age, and 11–50% in people >80 years [3]. Approximately 600 million people worldwide were classified as being over 60 years old in 2000. By 2025, this number is predicted to grow to 1.2 billion, with further increases to 2 billion as the years progress [4]. Based on the most conventional estimates, sarcopenia could affect more than 200 million people in 40 years’ time. In terms of mortality and morbidity, sarcopenia impacts older people greatly, causing substantial costs in terms of health care, disability, and morbidity [5,6]. Geriatricians and scientists in Europe and Asia have devised diagnostic criteria for sarcopenia that can be used worldwide as a clinical and public health methodology. An agreement on defining sarcopenia, as well as diagnostic criteria, was first proposed by the European Working Group on Sarcopenia for Older People (EWGSOP1) in 2010 [7]. Following this, an international sarcopenia definition was formulated by the International Working Group on Sarcopenia (IWGA) in 2011 [8]. In 2014, the Asian Working Group for Sarcopenia (AWGS1) released its regional guidelines to support research on sarcopenia in Asia [9]. Later, the Foundation for the National Institutes of Health (FINH) began its work on the sarcopenia project in 2014 [10,11]. Again a new version of the EWGSOP2 was published in 2018 to update the definition and diagnosis of sarcopenia [12]. Recently, sarcopenia research in Asia was promoted as part of revised guidelines by the Asian Working Group for Sarcopenia (AWGS2) in 2019 [13].

Sarcopenia is indicated by persons with a low muscle mass, weak handgrip strength, or slow walking speed as defined by EWGSOP criteria, but based on IWGS criteria, sarcopenia is defined as slow walking speed and reduced muscle mass in the body. There is only very limited agreement between IWGS and EWGSOP when defining sarcopenia, and its prevalence varies greatly when measuring skeletal muscle mass. Selecting the optimal cutoff values for handgrip strength, walking speed, and skeletal muscle indices, taking into account gender and ethnicity differences, is crucial for achieving universal diagnostic criteria for sarcopenia [14]. Sarcopenia is defined differently by the EWGSOP2 criteria than by EWGSOP1, AWGS, IWGA, and FNIH. A comparison of the risk factors associated with sarcopenia defined by EWGSOP2 with those defined by EWGSOP1, AWGS, IWGA, and FNIH does not reveal any consistent patterns. In light of this, prospective studies are needed in order to confirm the validity of EWGSOP2 or AWGS2 [15]. The WCHAT study, a trial of West China Health and Aging Trends in western China, provided baseline data for a study that was conducted in 2021 to investigate the prevalence and associated risk factors for sarcopenia in 4500 participants. Measurement of muscle mass was performed using bioimpedance analysis (BIA). A measurement of handgrip strength and measures of walking speed were also recorded. A variety of variables were collected, such as anthropometry measurements, lifestyle, chronic diseases, and blood tests. AWGS2 and IWGS provided independent correlations between serum albumin level and sarcopenia. EWGSOPT2 and FNIH both showed an independent association between vitamin D level and sarcopenia. AWGS1 significantly associated age, depressive status, body mass index (BMI), hemoglobin, vitamin D, and insulin level with sarcopenia, but AWGS2 did not associate any of these factors with sarcopenia. Various sarcopenic criteria are principally associated with low serum albumin levels and low vitamin D levels. Most risk factors associated with AWGS1 defining sarcopenia have not consistently been observed in AWGS2. In addition to such prospective studies, a multi-pronged approach for treating this disease is required to confirm the validity of the AWGS2 consensus [16].

**Figure 1:**
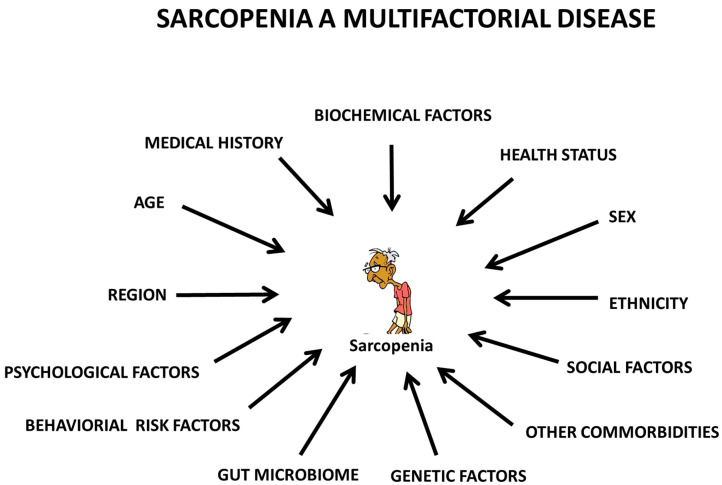
Sarcopenia associated with multiple factors including age, sex, region, ethnicity, medical history, health status, psychological factors, social factors, behavioral factors, gut microbiome, genetic factors, other comorbidities, and biochemical factors.

**Figure 2:**
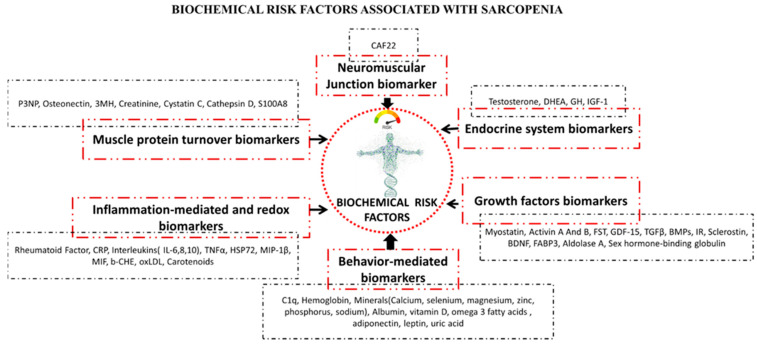
Biochemical risk factors are contributed by different pathways. Biomarkers associated with people with sarcopenia based on different pathways include neuromuscular junction biomarkers (CAF22); endocrine system biomarkers (T, DHEA, GH, IGF1); growth factors biomarkers (myostatin activin A and B, FST, GDF-15, TGFβ, BMPs, IR, sclerostin, BDNF, FABP3, aldolase A, sex hormone-binding globulin); behavioral-mediated biomarkers (C1q, hemoglobin, minerals (calcium, selenium, magnesium, zinc, phosphorus, sodium), albumin, vitamin D, omega 3 fatty acids, adiponectin, leptin, uric acid); inflammation-mediated and redox biomarkers (rheumatoid factor, CRP, interleukins (IL-6, 8, 10), TNFα, HSP72, MIP-1β, MIF, b-CHE, oxLDL, carotenoids); muscle protein turnover biomarkers (P3NP, osteonectin, 3MH, creatinine, cystatin C, cathepsin D, S100A8). CAF22, C-terminal agrin fragments 22; T, testosterone; DHEA, dehydroepiandrosterone; GH, growth hormone; IGF1, insulin-like growth factor-1; FST, follistatin; GDF-15, growth differentiation factor 15; TGFβ, transforming growth factor beta; BMPs, bone morphogenic proteins; IR, irisin; BDNF, brain-derived neurotrophic factor; FABP3, fatty acid binding protein 3; C1q, complement component 1q; TNFα, tumor necrosis factor α; HSP72, heat shock 70 kDa protein 1; MIP-1β, macrophage inflammatory protein 1β; MIF, macrophage migration inhibitory factor; b-CHE, butyryl-cholinesterase; oxLDL, oxidized low-density lipoprotein; P3NP, N-terminal peptide; 3MH, 3-methylhistidine.

As sarcopenia has serious and life-changing effects on older people, health professionals around the world need to synergize and collaborate to translate our growing body of knowledge into practical actions that will positively impact millions of older people globally. In the fourth decade of life, skeletal muscle mass and strength decline linearly. By the eighth decade of life, 50% of skeletal muscle mass is lost [17]. It is estimated that sarcopenia affects 5% to 13% of community-dwelling older people over 65 years. Those older than 80 and those who live in residential care or hospitals are more likely to be affected by this condition [18]. A person’s muscle mass decreases at a rate of about 8% per decade from the age of 50 until 70 years, where it is accelerated by about 15% per decade [19]. Nonetheless, many individuals continue to debate what the appropriate cutoff value is for diagnosing sarcopenia and, more practically, what the most effective screening tool should be [17]. A comparison of the risk factors associated with sarcopenia based on EWGSOP (1 and 2), AWGS (1 and 2), IWGS, and FNIH revealed no consistent patterns. Therefore, the identification of a single risk factor for sarcopenia is unreliable due to its “multifactorial” pathogenesis, with the involvement of a multitude of factors. The primary objective of this review was to compile information related to the multiple factors associated with sarcopenia based on studies outlined by the working organizations for this condition (Figure 1) to facilitate future studies. A further aim was to describe the pathophysiological mechanisms of sarcopenia, and in an attempt to determine the patient’s condition, a panel of biomarkers has been proposed that comprise six major pathways (Figure 2) involved in sarcopenia that may need to be evaluated and summarized for future diagnosis. This may help determine the biological pathways of the disease, contributing to further research. This may also contribute to establishing a uniform consensus for screening and defining the disease. Additionally, we discuss the impact of sarcopenia individually, socially, and financially. Finally, we emphasize that its impact during the COVID-19 pandemic may be exacerbated in older populations, which require scrutiny and investigation as a matter of urgency.

## 2. Methodology

A structured search strategy was conducted in PubMed and Google Scholar to search for publications in English using the search term “sarcopenia” in combination with one of the following keywords: “factors”, “epidemiology”, “ethnicity”, “health status”, “quality of life”, “symptoms”, “functional limitations”, “medical history”, “comorbidities”, “genetic factors”, “gut microbiota”, “gut microbiome”, “psychological factors”, “social factors”, “psychosocial factors”, “behavioral risk factors”, “biochemical factors”, “biomarkers”, “impacts”, “social impacts”, “individual impacts” and “financial impacts”. Furthermore, we also searched each biochemical biomarker mentioned in this review for the most updated related research. We focused on clinical trials, meta-analyses, and review articles. We did not include research related to sarcopenia management. The search was completed on 1 August 2021. When many similar articles were available, the most recent were used. Additional papers were identified from random searches and the reference lists of retrieved articles. No date restriction was put on the search so that a longitudinal “map” of the subject area could be obtained. These approaches resulted in a total of 159 articles for possible inclusion within this review.

## 3. Risk Factors for Sarcopenia

### 3.1. Epidemiology of Sarcopenia

#### 3.1.1. Region and Gender

A systematic review of studies revealed sarcopenia (EWGSOP1 criteria) to be prevalent in 1% (Finland) to 29% (Italy) of people over 50 years old; 10% (UK) for acute hospital-care settings, and from 14% (The Netherlands) to 33% (Italy) for long-term care institutions [20]. Pooled analysis of nine American studies of people aged 65 and over found that 5.3% of men and 13.3% of women had sarcopenia [21]. According to a study of approximately 4000 people residing in the community aged 65 and older in Hong Kong, 9.0% of subjects had baseline sarcopenia (EWGSOP1 criteria). The prevalence (EWGSOP1 criteria) rates in elderly community-living populations range between 1% and 29% and between 14% and 33% in long-term care populations [20]. Sarcopenia prevalence (AWGS criteria) in Asia ranges from 2.5% to 45.7% [9,22]. There are differences in the prevalence of sarcopenia among study populations by countries, by ages, by gender, by settings, and by measurement methods in Asia. The prevalence of sarcopenia in Asian populations varies substantially [9,23,24]. The existing Asian studies emanate from Japan, China, Taiwan, Korea, and Thailand.

Based on the current definition of sarcopenia, which also requires low muscle strength or inadequate physical performance, low muscle mass alone has a higher prevalence than the current definition [25]. According to a low muscle mass definition, the prevalence in older Asian adults ranges from 6.7% to 56.7% in men and 0.1% to 33.6% in women [9,26], while current definitions show 9% to 22.1% in men and 7.7% to 21.8% in women [9,27,28]. A meta-analysis of 58,404 elderly adults (2009–2016) was performed in 2017. According to the estimates, prevalence was 10% among men and 10% among women. In both genders, the prevalence was significantly higher among non-Asian individuals than Asians, when muscle mass was measured using BIA (20% compared with 10% in men; 11% in women) [29]. The prevalence of sarcopenia was reported to be 8.2% for men and 6.8% for women in a population-based cross-sectional study in Japan [30]. An important study in 2020 compared the prevalence of sarcopenia among Chinese community-dwelling older adults (*n* = 483, age 66.8 ± 4.4 years) based on all diagnostic criteria present on sarcopenia. The prevalence of EWGSOP2-defined sarcopenia (men: 6.5%; women: 3.3%) was lower than that defined by the EWGSOP1 (men: 22.3%; women: 11.7%), AWGS (men: 10.9%; women: 8.0%), and IWGS (men: 24.5%; women: 11.0%) criteria, but higher than FNIH criteria (men: 6.0%; women: 1.7%) [15].

#### 3.1.2. Ethnicity

There have been very few studies conducted investigating the prevalence of sarcopenia in different ethnicities. The prevalence of sarcopenia in adults (aged 18 and older) was analyzed using data from the National Health and Nutrition Examination Surveys from 1999 to 2004 in a study published in 2018. The prevalence of sarcopenia varies by sex and race or ethnicity: Hispanic (26.8% male, 27.2% female); non-Hispanic (NH) white (15.5% male, 15.1% female); NH black (8.6% male, 1.6% female); and other (16.5% male, 23.2% female) [31]. Another large-scale study (*n* = 10,325), from Louisiana compared the prevalence rates between whites, blacks, Asians, and Hispanics. In men, the rates of sarcopenia and sarcopenic obesity were 17.6% and 7.0%, respectively, and in women, the rates were 13.7% and 2.5%, respectively. The prevalence (AWGS criteria) of sarcopenia in individuals (*n* = 4500, age ≥ 50 years) from Yunnan, Guizhou, Sichuan, and Xinjiang provinces was reported. The prevalence of sarcopenia was 22.3% in Han, 18.2% in Tibetans, 11.8% in Qiang, 34.7% in Yi, and 26.7% in Huis [32].

### 3.2. Health Status

Health status is determined by the effect of disease on patient function, as described by patients through three components including the range of symptoms a patient is experiencing, their functional limits (physical and mental), and the quality of their life, which is defined by the discrepancy between their current and desired level of performance. Range of symptoms includes reduced skeletal muscle mass, reduced strength, and/or diminished physical performance. These are the main reasons older people are restricted in their daily activities, leading to disability, falls, hospitalization, fractures, or death [9,12,33]. Symptom-screening questionnaires including “Strength, assistance with walking, rising from a chair, climbing stairs, and falls” (SARC-F) by EWGSOP2 [12] and SARC-F combined with calf circumference (SARC-CalF) by AWGS2 are being used [13]. Handgrip test (to quantify muscle strength), chair stand test (to gauge lower extremity strength), gait speed test (to test 4 m usual walking speed), short physical performance battery (to test poor physical performance, including chair stand tests, standing balance, and walking speed), timed-up and go test (to indicate physical deficits by observing the time taken for a subject to rise from a chair, walk 3 m away and 3 m back to the chair, terminating the test in a sitting position) and the 400 m walk test (to assess physical inability to walk in a series of 20 × 20 m laps as quickly as possible, with a maximum of 2 min rest between each lap) are among the most common assessment methods used for assessing the symptoms of sarcopenia [33,34].

Apart from screening and assessing symptoms of sarcopenia, functional limits that include both mental and physical limitations are second important components of health status. In 2021, research examined the association between sarcopenia and its components (based on AWGS) with cognitive function (based on the Montreal Cognitive Assessment) in Chinese aging community dwellers (*n* = 428, age ≥ 80 years). The result revealed that sarcopenia had an overall prevalence of 35.5%, with 40.34% in men and 32.14% in women. Mild cognitive impairment was significantly associated with sarcopenia, low handgrip strength, and slow gait speed in community-dwelling elders. The prevalence of mild cognitive impairment was higher among sarcopenic older people than older people without sarcopenia [35]. Another study was conducted in 2020 among Thai community-dwelling older adults (*n* = 330, age 66.85 ± 5.54 years, 76.06% female) to explore the association of sarcopenia (AWGS criteria) with depression, cognitive performance, and physical activity (gait speed, muscle mass, and handgrip strength) reported 16.1% of participants had sarcopenia. Furthermore, advanced age, high depression scores, mild cognitive impairment, and low physical activity levels were significantly associated with sarcopenia after adjusting for age, sex, and educational level [36].

The third component, which is health-related quality of life (HRQoL), is one of the main factors in determining health status. Recently in 2021, a study was performed to examine the relationship between severity of sarcopenia and HRQoL in community-dwelling middle-aged people. They reported the independent association between probable sarcopenia and poor HRQoL, but not with confirmed or severe sarcopenia [37]. Sarcopenia and Quality of Life (SarQoL), a self-administered questionnaire to assess health-related quality of life in sarcopenia, has recently been developed. This questionnaire is valid, consistent, and reliable, which makes it suitable for clinical and research studies [38]. Another study from Belgium (*n* = 387) found that lower QoL was found for sarcopenic subjects compared with non-sarcopenic subjects by using the definitions of Cruz-Jentoft, Studenski, Fielding, and Morley [39]. Another study from the Korean National Health and Nutrition Examination Survey conducted between 2008 and 2011 (*n* = 4937, aged ≥ 60 years) measured health-related quality of life. They reported that sarcopenia was prevalent in 6.6% of Korean people (age ≥ 60 years), 11.1% in men, and 3.2% in women. They also concluded that findings differ between men and women but that there is a clear link between sarcopenia and poor health-related quality of life in older Korean populations [40].

### 3.3. Medical History/Comorbidities

A total of 20 of the 21 prognostic outcomes of tumor were found to be associated with sarcopenia in an umbrella review based on 30 meta-analyses. They reported that sarcopenia is significantly associated with adverse health outcomes, especially in the case of cancer patients and elderly populations. Additionally, sarcopenia was also associated with metabolic disorders, depression, and albuminuria [41]. A cross-sectional study conducted in Brazil, (*n* = 1078, age ≥ 60 years) defined by the EWGSOP, showed that diabetes mellitus (DM) was present in 36.87% of patients, with a greater incidence among sarcopenic patients. Female sex, advanced age, DM, cardiovascular problems, osteoporosis, BMI, waist circumference, triglycerides, and physical function were all associated with sarcopenia [42]. An analysis of 112 chronic liver disease patients (57 men and 55 women), including 40 cirrhotic patients (36%), examined the relationship between osteoporosis and sarcopenia. Among the elderly, 13% had sarcopenia, 17% had osteoporosis, and 65% had osteopenia. There was a significant and high incidence of osteoporosis in sarcopenic and cirrhotic patients [43]. In a further study, sarcopenia and obesity were linked to peptic ulcer disease (PUD). In this study, 7092 patients were divided into four categories: sarcopenic obesity (870), sarcopenic overweight (2676), non-sarcopenic overweight (2698), and non-sarcopenic overweight (848). Among these groups, the prevalence of PUD was 7.9%, 7.4%, 6.3%, and 3.8%, respectively. They concluded that increased risk of PUD was determined when muscle mass, fat mass, and obesity were considered together [44].

Furthermore, even neurological diseases need to be considered as an important risk factor. In an observational study, 104 Parkinson’s disease patients enrolled in a tertiary center and 330 non-Parkinson’s disease controls enrolled in a population-based cohort of individuals 65 years of age. Patients from a tertiary center who had Parkinson’s disease had a 55.8% prevalence of sarcopenia, while non-Parkinson’s disease controls had 8.2% prevalence. The prevalence of sarcopenia in the Parkinson’s disease community sample was 33.3%, and frailty was 22.2%. Both frailty and sarcopenia were found to be more common in Parkinson’s disease patients than in the general population and predicted a more detrimental outcome for patients [45]. The onset of dementia and sarcopenia was also evaluated using a meta-analysis and reported an increased prevalence of sarcopenia in dementia patients [46]. The study described a 78-year-old man who had a compromised cognitive capacity, waning strength in the lower extremities, and gait difficulties. In addition to sarcopenia and polyneuropathy, he was also diagnosed with Alzheimer’s disease. Chronic elevations of inflammatory markers were also detected. Researchers originally thought inflammation with aging, or “inflammaging”, contributed to multiple comorbid diseases. A multidisciplinary treatment program and comprehensive rehabilitation allowed him to ambulate again with only little to moderate assistance [47]. In this manner, various age-related disorders should be considered when an elderly patient has chronic low-grade inflammation and sarcopenia.

### 3.4. Genetic Factors

The genetic causes of sarcopenia are unclear, despite strong evidence that muscle phenotypes are inherited. Studies that use a genetic risk score for predicting sarcopenia risk are even rarer. Since each of the genes has only a small to moderate effect on sarcopenia risk, it is not necessary to identify the individual genes to understand genetic susceptibility. A polygenic model should be used to explain genetic variations in sarcopenia risk. In 2009, a study tested 379,319 eligible single nucleotide polymorphisms (SNPs) by genome-wide association scan (GWAS) in 1000 unrelated US whites and found that two SNPs, rs16892496 and rs7832552, within the **thyrotropin-releasing hormone receptor** (TRHR) gene were significantly associated with lean body mass (LBM). They also replicated the significant associations in three independent samples: (1) 2955 Chinese unrelated subjects, (2) 1488 unrelated US whites, and (3) 593 nuclear families comprising 1972 US whites. This study concluded that the TRHR gene is as an important gene for LBM variation [48]. In 2012, an overview of the major and representative molecular genetic studies was conducted, which focused on identifying genetic factors for sarcopenia. They reviewed human whole-genome linkage studies, quantitative trait loci mapping in animal models, newly reported GWAS, candidate gene association studies, DNA microarrays, and microRNA studies of skeletal muscle phenotypes such as sarcopenia. In the study, the authors reported that the **angiotensin I-converting enzyme I** (ACE), **myostatin** (MSTN), **alpha actinin 3** (ACTN3), **ciliary neurotrophic factor** (CNTF), and **vitamin D receptor** (VDR) genes associated with skeletal strength or mass in several studies The MSTN gene explained a large proportion of the variation in skeletal muscle traits, as corroborated by association studies, linkage studies, and expression studies. A possible importance could be attributed to the genes **insulin-like growth factor 1** (IGF1) and **interleukin-6** (IL-6), supported by evidence of linkage and association studies [49]. Another case control study was conducted on 175 Taiwanese community-dwelling patients with sarcopenia (56 severe sarcopenia, 63 sarcopenia, and 56 pre-sarcopenia) based on AWGS and 327 age- and gender-matched controls. They reported that **caveolin-1** (CAV1) may play an important role in the etiology of sarcopenia. They proposed that the A allele of Cav1 G14713A may serve as an pre-indicator for detection of severe sarcopenia and sarcopenia [50]. In 2020, a study using 2207 unrelated Caucasian subjects to assess the associations between lean mass and **fat mass and obesity-associated** (FTO) gene was performed by GWAS of lean mass index (LMI). They also replicated major findings in two replication samples including 38,292 unrelated Caucasians and 6004 unrelated Caucasians. They reported 29 SNPs in FTO significantly associated with sarcopenia [51]. Additionally, in 2020, another study based on genetic markers attempted to identify patients who were more likely to develop certain conditions or diseases, such as sarcopenia, or to develop joint problems. According to the traditional GWAS methodology, SNPs associated with muscle phenotypes and specific genes were chosen according to their *p*-values for muscle phenotype associations. They found **nudix hydrolase 3** (NUDT3) and **kruppel-like factor 5** (KLF5) for lean mass and **HLA-DQB1 antisense RNA 1** (HLA-DQB1-AS1) associated with hand grip strength as main genes to target for these phenotypes. The associated regulatory SNPs are rs464553, rs1028883, and rs3129753, respectively [52]. Later in 2021, in a subsequent research study, seven candidate gene variants were tested individually and together for their effect on sarcopenia risk. Kompetitive allele-specific PCR was used to genotype single nucleotide polymorphisms in candidate genes. As determined by the EWGSOPs diagnostic criteria, 190 older adults were classified as sarcopenic or nonsarcopenic. Sarcopenia was reported to be associated with **methylenetetrahydrofolate reductase** (MTHFR), **alpha-actinin-3** (ACTN3), and **nuclear respiratory factor 2** (NRF2) genotypes. Together, all three polymorphisms described 39% of the variation in sarcopenia risk inter-individually [53]. To treat muscle wasting diseases such as sarcopenia, researchers extensively studied the functions and structural properties of MSTN (encoded by the MSTN gene). MSTN is the most studied member of the TGF-β family and a negative regulator of skeletal muscle growth and development [54]. Another study suggested that the amount of **PR domain-containing 16** (PRDM16) gene could regulate brown adipose tissue, white adipose tissue, and muscle cell metabolism [55]. In 2021, a GWAS meta-analysis of low grip strength in European participants (age ≥ 60 years) from 22 studies yielding a combined sample of 254,894 individuals was conducted. They revealed that 15 genetic loci including **HLA class II histocompatibility antigen-DRB1 beta chain** (HLA-DRB1), **growth differentiation factor 5** (GDF5), **dymeclin** (DYM), **deleted in lymphocytic leukemia 1** (DLEU1), **solute carrier family 39 member 8** (SLC39A8), **RN7SK pseudogene 297** (RN7SKP297), **chromosome 12 open reading frame 60** (C12orf60), **retinoblastoma-binding protein 6** (RBBP6), **aldehyde dehydrogenase 1 family member A2** (ALDH1A2), **transforming growth factor alpha** (TGF α), **zinc finger and BTB domain-containing protein 38** (ZBTB38), **BR serine/threonine kinase 1** (BRSK1), **amine oxidase copper-containing 1** (AOC1), FTO **and zinc finger protein 678** (ZNF678) were associated with the EWGSOP definition of low grip strength and with two additional loci for the FNIH criteria, which uses a more strict cutoffs for muscle weakness. They reported two lead variants (ALDH1A2 and variants near FTO) that were previously not determined to be associated with anthropometric or musculoskeletal phenotypes in GWAS [56]. A very recent study in 2021 used the Gene Expression Omnibus (GEO) profiles of the National Center for Biotechnology Information for exploring associations between mRNA expressions of biomarkers and sarcopenia. They recruited (*n* = 408, age = muscle strength is an important heritable indicator of poor health linked to morbidity and mortality in older people. **Procollagen type III N-terminal peptide** (P3NP), **apelin and high-temperature requirement serine protease A1** are useful for diagnosis of sarcopenia in the clinical setting [57]. Several studies have been conducted on nematodes Caenorhabditis elegans and zebrafish to reveal the genetic basis of sarcopenia, but we have not examined those genes in this review [54,58].

### 3.5. Gut Microbiota

In older adults, changes in gut flora composition are associated with a progression of disease and frailty. Van Tongeren et al. [59] were the first researchers who reported for the first time that frailty is associated with microbiota composition in the gut. In particular, a decline in the amount of **Bacterioides/Prevotella**, **Lactobacilli**, and **Faecalibacterium prausnitzii** and an increase in the proportion of **Atopobium, Ruminococcus**, and **Enterobacteriacae** were reported in subjects with rate-producing bacteria, suggesting that butyrate plays an important role in strengthening the junctions between intestinal cells and in preventing the spread of microbes [60]. In addition, a reduced level of inflammation may contribute to the maintenance of muscle tissue [61]. In the ELDERMET study (established in 2007 to investigate the role of intestinal microbiota as an agent and indicator of health in 161 Irish subjects aged over 65 years), the butyrate-producing bacteria were linked directly to functional capacity and health status in older community-dwelling adults [62]. Another study published in *Nature* in 2019, analyzed the gut microbiota, metabolic characteristics, and systemic inflammation among older adults with/without physical frailty and sarcopenia (PF&S). Community dwellers (*n* = 35; 18 with PF&S, and 17 nonPF&S, age ≥ 65 years) were enrolled. Their model correctly identified 91.7% of participants with PF&S and 87.5% of controls. PF&S subjects showed lower circulating levels of threonine and MIP 1α and higher serum concentrations of aspartic acid. Additionally, increased abundance of **Ruminococcus** and **Oscillospira** and decreased abundance of **Christensenellaceae** and **Barnesiellaceae** compared with controls was reported [63]. In 2020, a review compiled gut microbiota in relation to sarcopenia and also highlighted the relationship between the abundance of specific intestinal bacteria, serum levels of specific inflammatory biomolecules, and metabolic markers, suggesting the existence of an additional pathway through which changes in gut microbiota may impact on PF&S pathophysiology [63,64]. In research published in 2021, fecal samples of 60 healthy controls and 27 sarcopenic (case and precase) individuals were collected and analyzed. They reported an overall reduction in microbial diversity in case and precase samples. The genera **Lachnospira**, **Roseburia**, **Fusicantenibacter, Lachnoclostridum,** and **Eubacterium**—known butyrate producers—were reported as significantly less abundant in case and precase subjects, whereas **Lactobacillus** was reported to be more abundant in case and precase subjects. Analysis of the intestinal microbiota revealed structural and functional changes potentially contributing to skeletal muscle loss and function in sarcopenia [65].

### 3.6. Psychosocial Factors

The importance of psychosocial factors cannot be underestimated. A study demonstrated that self-efficacy, social isolation, fear of falls, resilience, depression, and social networks directly influence sarcopenia, and they indirectly influence sarcopenia through lifestyle factors. [66]. Psychosocial factors play a role in the origin of nutritional deficiency, and research has highlighted that individuals who eat alone [67] or have narrow social networks [68] are more expected to have malnutrition and to suffer from depression. For this reason, to assess nutritional deficiencies among the elderly, a tool called Mini Nutritional Assessment (MNA) was developed that includes psychosocial factors as well as nutritional measures [69,70]. According to AWGS2, most studies have found that nutritional and psychosocial factors individually contribute to sarcopenia, but a recent study investigated the effects of both factors simultaneously in 1019 older adults. “Probable sarcopenia (PS)” was characterized by low handgrip strength and reduced physical performance. The psychosocial factors including self-efficacy, fear of falling, social isolation, and social capital were examined. There were 426 men (41.8%) and 593 women (58.2%), with mean ages of 72.1 ± 7.6 years. A total of 29.8% of people in the study lived alone, 65.9% did not have an education beyond middle school, and 85.9% did not make more than 2 million Korean won salary per month. One aspect of PS in elderly adults is physical performance, which is affected by a variety of psychosocial factors [71]. Self-efficacy is allied with limitations in physical function [72] and gait speed [73]. Self-efficacy is known to mediate the relationship between lifestyle, including physical activity and functional limitations in the elderly, and is an important factor in lifestyle improvement activities such as exercise and nutrition [74,75]. Thus, improving physical function requires a cognitive approach aimed at increasing self-efficacy [76,77]. Sedentary behavior and physical inactivity are associated with falling fear [78], which deleteriously affects physical functioning among the elderly [79]. In order to reduce a fear of falling, increasing muscle strength and balance will be necessary to improve self-efficacy and promote independence. Sarcopenia, depressive symptoms, and social support were also examined in another study concerning self-efficacy for exercise. Muscle mechanography (MM) was also tested against traditional muscle function and strength tests for convergent validity. The development of greater muscle mass, strength, and function was shown to be associated with a high self-efficacy score, no depressive symptoms, and strong social support. Additionally, all tested measures of muscle function and strength were found to be valid [80]. In older adults, sarcopenia is one of the major causes of disability. In a study, muscle mass, strength, and function and their relationship with depressive symptoms, social support and exercise self-efficacy were examined in 31 residential care apartment complex (RCAC) residents. Interestingly, according to studies of community-dwelling older adults, residents of the RCACs had less muscle mass, strength, and function. It was shown that those who were self-efficacious, without depression symptoms and with powerful social support, had numerically greater muscle mass, strength, and function [81].

### 3.7. Behavioral Risk Factors

Sarcopenia is a condition where muscles lose mass and function as age proceeds due to lifestyle habits that include poor nutrition, physical inactivity, lack of exercise, alcohol consumption, and tobacco use. Sarcopenia is predicted to become more prevalent and costly as life expectancies increase worldwide. We must consider our lifestyle habits in order to delay and treat sarcopenia. Lifestyle factors can reduce the progression of sarcopenia far more than physiological or systemic changes as we age. Thus, educating the public about the importance of an active lifestyle in old age and its effects on skeletal muscle status is of great importance in the management of sarcopenia [82]. In Taiwan, 1068 older adults (>65 years) were surveyed via telephone to assess behavioral risk factors. Self-reported daily lifestyle behaviors (such as food intake and exercise) as well as sarcopenia (measured using the SARC-F) and personal characteristics were investigated in this study. Researchers found that older adults who chose unbalanced foods, did not engage in adequate physical activity, and sat for long periods of time had higher risks of sarcopenia. Sleep duration did not show a significant association. They concluded that as far as health behaviors were concerned, a poor diet (six nutrients), not getting enough physical activity (150 min/week), and prolonged sitting (≥7 h/day) may lead to sarcopenia in older adults. Interventions for the prevention of sarcopenia should aim to promote a balanced food selection, enough physical activity, and reducing sitting time in older adults [83]. The percentage of European middle-aged and older adults with probable sarcopenia ranged from 4.82% to 7.40% based on muscle strength, whereas the percentage of those with sarcopenia was minimal. In general, being older, sedentary, obese, physically inactive, and eating junk food resulted in an elevated risk of having low muscle mass, low muscle strength, or low functional performance—all factors connected with sarcopenia [84]. A decrease in food intake, particularly protein intake, due to lifestyle choices [85], sedentary behavior, physical inactivity, lifelong tobacco use, and alcohol abuse have all been associated with a high risk of sarcopenia [86]. Furthermore, people who spend long periods of time in bed and are immobile lose muscle mass and experience weight loss as they age [87].

### 3.8. Biochemical Factors

A number of complex biochemical interactions account for skeletal muscle changes as we age, including changes to neuromuscular junctions, endocrine systems, growth factors, and muscle protein turnover and metabolic dysregulations. As sarcopenia can be multifactorial in nature and involves many pathways, it is impossible to identify a single biomarker for the disease [88]. Here, we review a number of interesting studies and trials that have evaluated biomarkers associated with people with sarcopenia based on a variety of pathways (Figure 2):

#### 3.8.1. Neuromuscular Junction (NMJs) Biomarkers

One of the hallmarks of sarcopenia is the detonation of the NMJ [89]. Some studies suggest that the number of circulating **C-terminal agrin fragments** (CAF) is significantly higher in the sarcopenic population than in the non-sarcopenic population [90,91]. As an integral component of the neuromuscular junction, agrin aggregates acetylcholine receptors at the postsynaptic terminal. In sarcopenia, and in other catabolic diseases, agrin is cleaved into CAF22 via proteolytic cleavage, causing NMJ dysfunction. CAF22 levels have recently been reported to rise with sarcopenia in patients suffering from pulmonary diseases [92]. Further to this, accelerated sarcopenic patients consistently had higher CAF22 serum levels than did healthy controls. In individual cohorts of participants, serum CAF22 levels were not correlated with SPPB (Short Physical Performance Battery) or SARC-F (questionnaire, a screening tool that can be rapidly implemented by clinicians to identify probable sarcopenic patients, including deficiencies in strength, walking, rising from a chair, climbing stairs, and experiencing falls), two tools that can rapidly identify sarcopenic patients [93].

#### 3.8.2. Endocrine System Biomarkers

**Testosterone** (T) is a steroid hormone produced mainly by the testes, ovaries, and the adrenal cortex, which stimulates the development of secondary sexual characteristics in men. It also synthesizes muscle protein [94]. In a review of the literature, T supplementation was found to reduce the loss of muscle mass and grip strength [95]. While epidemiological studies have shown inconsistent results, lower T levels have been linked to decreased muscle mass and function [96,97,98,99]. In a recent study with 396,707 participants (68.8% of whom were women, aged 38 to 73 years) with sarcopenia (EWGSOP2 criteria), higher T concentrations were associated with sarcopenia in women only [100]. **Dehydroepiandrosterone** (DHEA) is a hormone produced in the adrenal gland; especially due to its unique age-related effects, this precursor of androgen is often considered a senescence marker. It declines as a result of the loss in muscle mass [101] and strength [102]. Additionally, higher levels of DHEA are linked to fewer falls [103]. Muscle mass is also affected by DHEA [104]. **Growth hormone** (GH; produced by the pituitary gland located at the base of the brain) concentrations decline gradually with age at the same rate as T, but more importantly, its production drops between 5- and 20-fold below that of young adults after the age of 30 years. Deficiencies in GH result in muscle loss but not in muscle strength. Insulin resistance or insulin deficiency accelerates the development of sarcopenia [105]. **IGF-1** is produced by the liver primarily, along with the target tissues in an autocrine and paracrine manner. In a recent cross-sectional study, researchers examined elderly patients (*n* = 3276). A body composition survey, grip strength, and speed measurements were taken. Sarcopenia (AWGS criteria) in the older population was associated with GH and IGF-1. The reduction in skeletal muscle mass was linked to IGF-1, mechanical growth factor, and BMI, as well as gender [106]. The lowest IGF-1 concentrations were linked with sarcopenia in men and women in another large study of 396,707 participants (68.8% women, ages 38 to 73 years) from the UK Biobank (EWGSOP2 criteria) [100]. In another study, community-dwelling adults from Seoul (*n* = 96, age ≥ 60 years) were assessed for sarcopenia (AWGS criteria) and reported lower serum IGF-1 levels in sarcopenic patients compared with controls [107].

#### 3.8.3. Growth Factors Biomarkers

There is a theory that sarcopenia is caused by an imbalance between growth-enhancing and growth-suppressing factors, favoring the latter. **MSTN** is produced by myocytes and is released into the body to inhibit muscle cell growth. It is a possible candidate for muscle mass negative regulation. Its expression in sarcopenia may be identified only in satellite cells, rather than throughout all muscles [108]. Despite not being a feasible biomarker in isolation, a recent review concluded that MSTN could be a valuable contributor to a recently suggested panel of muscle wasting biomarkers [109]. Moreover, Laurent et al. [110] in a letter to the editor raised concerns that sarcopenia and frailty often appear together but are separate disorders and asserted that there must be a clearer correlation between MSTN and each condition separately. A second claim has been made that MSTN’s influence on circulating versus autocrine/local hormones needs to be better understood [110]. **Activin A and B**, are members of the transforming growth factors (TGF)β superfamily and may have an important role in regulating muscle mass. In cancer-induced cachexia, they are 100 times more effective than MSTN in causing muscle wasting in some mouse models of the disease [111]. Sarcopenia patients, however, have not been investigated extensively. **Follistatin** (FST) is an autocrine glycoprotein that is expressed in virtually every tissue in higher animals. It inhibits MSTN, activin A, and TGFβ and consequently enhances muscle progenitor cell-dependent myogenesis [112]. Recent research in 2020, however, concluded that sarcopenic prevalence of 56.4% was found among patients with hepatocellular carcinoma and that serum FST level was an independent factor for poor survival [113]. **GDF-15** is expressed at low levels in many organs but is upregulated by injury to the liver, kidney, heart, and lungs. In patients hospitalized in intensive care, the expression of GDF-15 was associated with a reduction in the expression of some microRNAs that contribute to muscle growth [114]. An analysis of cross-sectional data (*n* = 929) as well as two-year prospective data (*n* = 788, age 70–84 years) in the Korean Frailty and Aging Cohort Study was conducted recently. The participants at baseline had a sarcopenic condition of 16.6%. Sarcopenia was associated with higher median GDF-15 concentrations than non-sarcopenia patients. After a 2-year follow-up, researchers found elevated GDF-15 linked with widespread sarcopenia, but the prediction of incident sarcopenia could not be made [115]. GDF-15 must be investigated further to understand its pathophysiological effects on sarcopenia. Every lineage of white blood cells produces TGFβ. It has been shown to inhibit myogenesis [116], but it is unclear how it contributes to sarcopenia. **Bone morphogenic proteins** (BMPs) are growth factors also called cytokines and metabologens. A recent study revealed that BMPs directly compete with MSTN/activin/TGFβ in skeletal muscle, leading to increases in muscle mass [116], although their role in sarcopenia is not fully understood. **Irisin** (IR) is produced and expressed by adipose tissue and muscle in association with obesity and insulin resistance. It has been investigated in individual cohorts of healthy controls and chronic heart failure and COPD patients (*n* = 81–87/group, aged 55–74 years); the categories with sarcopenia (EWGSOP criteria) were found to be associated with low levels of IR [93]. Sarcopenia was found in 73 (39%) of 187 patients with liver cirrhosis in a recent study (*n* = 187). The findings revealed that sarcopenia patients had lower IR concentrations than nonsarcopenic patients [117]. A total of 715 Koreans living in the community were evaluated for anthropometrics, body composition, and sarcopenia-related parameters proposed by the AWGS, as well as serum IR levels. Researchers concluded that low levels of circulating IR are sensitive indicators of muscular weakness and atrophy and could be used to predict sarcopenia and monitor age-related changes in muscle fibers [118]. **Sclerostin** is a protein secreted by bones (osteocytes), and it inhibits bone growth. In an evaluation of 92 patients on hemodialysis examining the prevalence, levels of chronic sarcopenia, and metabolic parameters associated with the disease, results suggested that hemodialysis patients with diabetes had higher serum sclerostin concentrations and that these were inversely correlated with muscle mass [119]. **BDNF** is a neurotrophic factor that helps neurons develop, mature, and survive, and it shows a neuroprotective effect in adverse environments. In a retrospective cross-sectional study, Japanese patients (*n* = 20, aged > 65 years) who were undergoing maintenance hemodialysis over 6 months were assessed. The study concluded that BDNF deficiency can result in decreased physical performance and a higher likelihood of severe sarcopenia and frailty among Japanese maintenance hemodialysis patients. Loss of BDNF reduces the motor end plate volume without affecting the integrity of the NMJ. Slowing muscle function and increasing resistance to fatigue caused by contractions are associated with these morphological changes. On the other hand, BDNF overexpression promotes the expression of fast muscle-type genes and increases the number of glycolytic fibers. Based on these findings, BDNF appears to be important for fiber type specification and may be a therapeutic target for diseases of the muscle [120]. **Fatty acid binding protein 3** (FABP3) participates in lipid transport, storage, signal transduction, oxidation, and transcriptional regulation. A group of male healthy controls and patients with chronic heart failure and chronic obstructive pulmonary disease (*n* = 81–87 per group, aged 55–74 years) were identified for sarcopenia (EWGSOP criteria). The study concluded that healthy controls, chronic heart failure patients, and COPD patients with sarcopenia status had higher levels of FABP3 in their individual cohorts [93]. **Aldolase A** is a glycolytic enzyme that catalyzes fructose-1,6-bisphosphate to glyceraldehyde-3-phosphate and dihydroxyacetone phosphate. Studying aldolase A levels with MS spectrometry analysis, researchers found that a sarcopenia group had 4.2-fold higher levels than the control group. ELISA and Western blotting did not detect differential expression of full-length aldolase A, but fragments of the protein were detected as being differentially expressed [121]. **Sex hormone-binding globulin** is a glycoprotein involved in binding androgens and estrogens. A recent UK Biobank study (*n* = 396,707) reported higher sex hormone-binding globulin concentrations in both men and women with higher sarcopenia (EWGSOP2 criteria) and even found an association [100].

#### 3.8.4. Muscle Protein Turnover Biomarkers

**N-terminal peptide** (P3NP) is the amino terminal peptide of type III procollagen, and it is released when type III collagen is synthesized and deposited. In a study, researchers recruited healthy male controls as well as people with chronic heart failure and chronic obstructive pulmonary disease (*n* = 81–87/group), aged 55–74 years based on EWGOS. They reported that sarcopenia status was associated with higher levels of P3NP and **osteonectin** (glycoprotein binds calcium to bone) in the individual cohorts of healthy controls, chronic heart failure, and chronic obstructive pulmonary disease patients [93]. In a further study, community-dwelling adults from Seoul (*n* = 96, age ≥ 60 years) were recruited. Sarcopenia was identified based on AWGS. Higher serum osteonectin levels in sarcopenic patients were reported compared with controls [107]. The amino acid **3-methylhistidine** (3MH) is found in actin and myosin. In a cross-sectional study of community-dwelling individuals (>65 years of age), plasma 3MH, 3MH/creatinine, and 3MH/estimated glomerular filtration rate were correlated with frailty status. In the context of the FRAILOMIC initiative, 360 participants from two French cohorts were categorized into robust, pre-frail, and frail using Fried’s frailty criteria. According to their research, 3MH, 3MH/creatinine, and 3MH/estimated glomerular filtration rate in plasma may be useful biomarkers for identifying frail individuals or those at higher risk of becoming frail [122]. There is still a need for further research, especially longitudinal research. **Creatinine** is a waste product formed by the normal wear and tear of muscles. Researchers from a study with 396,707 participants (68.8% women, ages 38 to 73) from the UK Biobank reported that sarcopenia was associated with, respectively, lower creatinine levels and higher **cystatin C** (the kidneys filter cystatin C from the blood and break it down at a constant rate) levels [100]. **Cathepsin D** is a ubiquitous lysosomal aspartic endo-protease. An immunoassay study found that the level of cathepsin D in the serum of patients with sarcopenia was higher than in normal people. Gait speed and cathepsin D levels in serum showed an inverse relationship. In order to improve diagnostic performance, a predictive model including cathepsin D, age, and BMI was developed (AUC = 0.908) [123]. According to EWGSOP1, 19 sarcopenic subjects and 20 controls were selected based on age, gender, and sarcopenia. Their MS analysis revealed that the sarcopenia group showed 2-fold higher cathepsin D levels and 4.2-fold higher **S100A8** levels than the control group. S100A8 are calcium- and zinc-binding proteins that regulate inflammation and immunity. Moreover, Western blot tests showed that sarcopenia patients had an enhanced detection of S100A8 [121].

#### 3.8.5. Behavior-Mediated Pathways Biomarkers

A variety of behavioral factors contribute to sarcopenia, including eating habits, obesity, and physical activity levels. Sarcopenia is well known to be associated with low physical activity and aging-induced increases in **complement component 1q** (C1q), which is involved in the complement system, a part of the innate immune system. A cross-sectional study analyzed the effect of serum C1q level on muscle mass and strength in 131 healthy participants, aged 20–81 years. A cross-sectional study found that serum C1q levels increased with aging and were negatively related to muscle density and strength. In this regard, C1q may be a novel indicator of sarcopenia, as it reflects the loss of muscle mass [124]. **Hemoglobin** transports oxygen through the blood. In a longitudinal aging study, 730 participants’ data were assessed for sarcopenia (AWGS2 criteria) and anemia (WHO criteria). The study found that 16.2% of patients were anemic, and 8.5% were sarcopenic. The higher the hemoglobin level, the faster the gait and the stronger the grip were observed. A high level of anemia was significantly linked with sarcopenia, slowness, and weakness. In men and those with a high disease burden, anemia and sarcopenia appeared to correlate more strongly. The authors concluded that anemia was found to be a risk factor for sarcopenia in older adults [125]. Regarding **minerals**, the prevalence of sarcopenia and/or the role of dietary mineral intake or mineral serum concentration was reviewed in a systematic review conducted in 2016 on healthy or frail older adults (average age ≥65 years). The results showed that serum **calcium** intake and **selenium** were significantly associated with muscle mass and that selenium, **magnesium**, **zinc**, and iron intake were positively associated with physical performance in the elderly. Furthermore, selenium, magnesium, calcium, and **phosphorus** intake were correlated with the prevalence of sarcopenia. The analysis concluded that magnesium, selenium, and calcium seem to be the most promising biomarkers based on observational studies [126]. **Albumin** is the most abundant circulating globular protein found in plasma. A study with total of 396,707 (68.8% women, age 38 to 73 years) participants from UK Biobank (EWGSOP2 criteria), reported that higher concentrations of albumin and **sodium** were associated with sarcopenia in both men and women, whereas lower values of **vitamin D** (fat-soluble secosteroids that increase intestinal absorption of calcium, magnesium, and phosphate) were associated with sarcopenia only in men [100]. Only a few studies specifically analyzed vitamin D status, sarcopenia, and functional performance of the oldest-old (above 85 years) [127]. There is a need for further research on the effects of vitamin D on muscle and skeletal health among adults in this age group [128]. Clinical trials have investigated the association between selenium, magnesium, and **omega 3 fatty acids** (polyunsaturated fatty acids) with physical activity and muscle performance in elderly individuals taking the supplements and consuming the foods containing these nutrients. Observation of the Mediterranean diet and higher intake of fruits and vegetables has been associated with better physical performance and protection from sarcopenia, muscle wasting, and the onset of frailty [129]. **Adiponectin** performs anti-inflammatory, anti-fibrotic, and antioxidant effects through its secretion by adipocytes. Researchers reported that sarcopenic participants had adiponectin levels that were significantly higher than those of their controls in a meta-analysis of seven studies. Sarcopenic individuals (AWGS criteria) were reported to have higher adiponectin levels in subgroup analyses in Asians, with a significant influence of female gender [130]. **Leptin** is predominantly produced by adipocytes and enterocytes in the small intestine; it regulates energy balance by suppressing hunger, which then reduces fat storage in adipocytes. In a study, 4063 older adults aged 60 years and over were enrolled from the NHANES III database. The study found that SMI and BMI were negatively correlated with serum leptin levels [131]. Higher handgrip strength was associated with higher plasma **uric acid** concentrations in older men and women [132]. Among 396,707 (68.8% women, ages 38 to 73 years) participants in the UK Biobank, sarcopenia was associated with urea, but only among women [100].

#### 3.8.6. Inflammation-Mediated and Redox Pathways Biomarkers

In 2020, a systematic review and meta-analysis was published for exploring the association between inflammation markers and muscle mass and strength. Overall, 168 articles, *n* = 89,194, were included. Higher levels of **C-reactive protein** (CRP), **tumor necrosis factor** (TNF)α, and IL-6 were associated with knee extension strength, muscle and lower handgrip strength, and muscle mass. A higher level of systemic inflammatory markers was associated with a decrease in muscle strength and mass over time [133]. Below we have compiled the studies that have investigated inflammatory/redox pathway biomarkers in association with sarcopenia. **Rheumatoid factor** is an immune system protein that may attack healthy cells in the body. A total of 240 Japanese patients with rheumatoid arthritis aged 65 years and older were studied according to AWGS criteria. Researchers found that patients with rheumatoid arthritis were more likely to suffer from sarcopenia based on age, BMI, C-reactive protein, and hip bone density [134]. Additionally, a study with a total of 396,707 (68.8% women, age 38 to 73 years) participants from UK Biobank defined by EWGSOP2 criteria reported that higher concentrations of rheumatoid factor and CRP is produced by the liver and increases during inflammation. CRP has been found to be associated with sarcopenia in both men and women [100]. **Interleukins** (IL) are a group of cytokines. In 2017, 17 studies with a total of 11,249 participants (3072 with sarcopenia and 8177 without) were meta-analyzed [135]. The authors noted no significant difference between sarcopenia patients and controls regarding serum IL-6 levels. IL-6 functions both as an anti-inflammatory myokine and a pro-inflammatory cytokine. A further study, however, showed that patients with obesity and diabetes who develop sarcopenic obesity as they age have persistent and markedly elevated levels of pro-inflammatory cytokines such as IL-6 [136], indicating subtle relationships between endocrine and metabolic phenomena and inflammatory aging. Chronic low-grade inflammation caused by IL-6 is a major cause of sarcopenia and part of the aging process. The activity of IL-6 during physiological conditions is limited by the duration of the injury, but the signaling of IL-6 causes chronic low-grade inflammation [137]. Further research on community-dwelling adults from Seoul (*n* = 96, age ≥ 60 years) was conducted. Sarcopenia was identified based on AWGS. They reported higher serum IL-6 levels in sarcopenic patients as compared with control [107]. The same meta-analysis found that sarcopenic patients’ TNFα (which is the cell-signaling protein of the immune system) levels were not higher than their control counterparts. According to the results, sarcopenia may be associated with elevated blood CRP levels; further studies are needed to clarify the link [135]. **Heat shock 70 kDa protein 1**(HSP72) is a protein that under stressful conditions facilitates the folding of newly synthesized proteins, facilitates the translocation of precursor proteins into organelles, and helps in the degradation of damaged proteins. Researchers tested plasma levels of HSP72, serum CRP, IL-6, TNFα, and other biomedical parameters in blood samples from both men and women (*n* = 665). Having higher Hsp72 levels in the plasma may be a useful biomarker of sarcopenia in the elderly since it was associated with lower muscle mass, weak grip strength, and slower walking speed [138]. As part of the study on biomarkers associated with sarcopenia and physical frailty in elderly people, 200 community dwellers, 100 with physical frailty and sarcopenia and 100 without the condition were recruited. Among 74 biomarkers of frailty and sarcopenia, aspartic acid and HSP72 showed higher levels in serum, and **macrophage inflammatory protein 1β** (MIP-1β) (a chemoattractant, as well as a neutrophil stimulator) had lower levels, with peculiar gender differences [139]. **Macrophage migration inhibitory factor** (MIF) is a key regulator of innate immunity that is found in virtually all cells. Males aged 55 to 74 years from healthy controls and patients with chronic heart failure and chronic obstructive pulmonary disease (*n* = 81–87/group) were recruited to participate in a study. Using the EWGOS recommendations, sarcopenia was clinically diagnosed. The MIF levels were higher in healthy controls as well as in chronic heart failure and chronic obstructive pulmonary disease patients who were classified as having sarcopenia (SPPB score of 8 or SARC-F score of 4) in general [93]. Higher serum MIF levels in sarcopenic patients were also reported compared with controls among community-dwelling adults from Seoul (*n* = 96, age ≥ 60 years) based on AWGS [107].

Another observational study indicated an association between chemotherapy-induced sarcopenia and the inflammatory markers HsCRP, IL-8, and TNF-α. In patients newly diagnosed with non-metastatic cancer, chemotherapy-induced sarcopenia may be exacerbated by inflammation [64,140]. **IL-10** is an anti-inflammatory, immunomodulatory cytokine responsible for regulating mucosal inflammation. IL-10-null mice, a rodent model of chronic inflammation and frailty with severe mitochondrial damage, were shown to have abnormal autophagosome formation in their skeletal muscle [141]. Inflammation is likely connected with muscle decline by mitochondrial DNA released from damaged organelles, which circulates in the bloodstream [85]. According to a study recently published, mitochondrial dysfunction contributes to sarcopenia’s pathogenetic mechanisms [142]. **Butyryl-cholinesterase** (b-CHE) enzyme is synthesized in the liver and hydrolyzes many different choline-based esters. A study demonstrated that b-CHE levels, which are routine markers of chronic inflammation and malnutrition, are linearly related to grip strength and muscular mass among elderly people. A total of 337 elderly subjects (mean age: 76.2 ± 6.7 years) were recruited for comprehensive geriatric assessment, and the study reported that b-CHE levels were lower in sarcopenic than in nonsarcopenic elderly subjects. Both men and women were found to have linear correlations between b-CHE and grip strength and muscular mass using linear regression analysis [143]. **Oxidized low-density lipoprotein** (oxLDL) promotes inflammatory processes and foam cell formation and is associated with atherosclerosis. A study included 65 women (age 67 ± 7 years) and 9 men (age 71 ± 8 years) from a rural village in Japan. Multiple linear regression analysis showed that changes in high-density lipoprotein and malondialdehyde-modified low-density lipoprotein (MDA-LDL/LDL-C) ratio were significantly and independently associated with changes in hand grip strength. Furthermore, in data stratified by gender, change in the MDA-LDL/LDL-C ratio was significantly and similarly associated with change in hand grip strength in women only [144]. **Carotenoids** are molecules that quench singlet oxygen and neutralize free radicals. A recent study of older people in the community demonstrated that low serum/plasma carotenoids are linked to decrease skeletal muscle strength as well as reduced walking ability. It is well known that a diet high in fruits and vegetables reduces inflammation, hypertension, diabetes, cardiovascular disease, and premature death. These observations agree with numerous studies that demonstrate this relationship [145]. In the Women’s Health and Aging Studies, 669 community-dwelling adults aged 70 to 79 years with no disabilities underwent cross-sectional analyses. Strength measures related independently to higher amounts of carotenoid and alpha-tocopherol. The findings were consistent with the hypothesis that oxidative stress causes sarcopenia in elderly individuals, but further longitudinal and interventional studies must be conducted to establish causality [146].

## 4. Impact of Sarcopenia

### 4.1. Impact at the Individual Level

Sarcopenia is characterized by decreased mobility, reduced muscle performance, and impaired metabolic health. Furthermore, sarcopenia is related to decreased locomotion ability, resting energy expenditure, nonstructural free-living physical activity, and increased fat mass, all of which have been linked to obesity and metabolic disorders. After the fifth decade of life, there appears to be an accelerated decline in muscle fiber size and number. Muscle loss is mild before age 50 years (<10%), but it increases to 30–40% between the age of 50 and 80 years [147]. Sarcopenia affects many aspects of one’s life, including disability and debilitating health problems such as osteoporosis, dyslipidemia, cardiovascular disease, metabolic syndrome, and immunosuppression [148]. The death rate for sarcopenic patients is 4 times higher than for non-sarcopenics. No significant difference in the results was seen depending on the setting of the participants (community-dwelling versus hospitalized subjects versus nursing home residents) or the length of follow-up. Age only influenced the results; as expected, subjects over the age of 79 years were more likely to be affected by sarcopenia. One recent meta-analysis attempted to determine the association between sarcopenia and mortality; however, the authors focused on specific definitions of sarcopenia and excluded studies that used muscle mass as the sole measure [149]. However, they also found that sarcopenic subjects had a higher mortality rate than non-sarcopenic subjects, with a hazard ratio of 1.87 [150].

### 4.2. Impact at the Social Level during the COVID-19 Pandemic

All aspects of society have been impacted by the COVID-19 pandemic, and this will continue to occur. Physical inactivity, a disruption in eating habits, stress, and irregular sleep patterns may put older people at greater risk of sarcopenia, which is associated with diminished overall quality of life and reduced mobility, as well as the development of several lifestyle-related diseases. Many individuals hospitalized by COVID-19 will also suffer from some degree of muscle weakness requiring some form of rehabilitation to recover lost strength and function [151]. Sarcopenia does not yet have a consensus operational definition. Public health outcomes related to this age-related condition include falls, fractures, hospitalizations, institutionalization, and mortality, among others. Consequently, these consequences directly increase personal, social, and health care system costs and will likely continue to do so as the population ages. Exercise programs that focus on improving muscle mass and function may be vital in reducing sarcopenia. In a systematic review published in 2019, de Mello et al. examined the effects of physical exercise programs versus no exercise intervention on sarcopenia features and its determinants in sarcopenic elders [152]. The aim of this study was to evaluate the effect of a six-month home-based resistance-training program on muscle health and physical performance in healthy older subjects during the unique condition of home confinement caused by the COVID-19 pandemic. The home-based resistance-training program during the lockdown period, caused by the COVID-19 outbreak, determined only within-group improvement in lower limb muscle strength but not in muscle mass and composition in older subjects [153]. In recent years, many older people have been affected by sarcopenia and its associated disabilities and that has created a medical and social challenge to develop effective and accessible preventive interventions [25,154]. Hence, there is a role for smart technologies and devices to promote remote coaching models during coronavirus pandemic because they may have the potential to provide an affordable alternative to coach-based exercise prescription. Exercising using a smartphone application improved cardiorespiratory fitness, body composition, cholesterol profiles, and psychological outcomes in people living with HIV in a 16-week protocol consisting of moderate physical activity three times per week, which included an initial coach-supervised period of 4 weeks, followed by 12 weeks in which participants trained independently [155].

### 4.3. Impact at the Financial Level

Patients with sarcopenia have higher levels of comorbidity, which results in higher costs. According to research, sarcopenia is independently linked to higher costs in the hospital environment. Patients with this condition are required to pay an additional 52.7% in hospitalization costs (58.5% for those over 65 and 34% for those older than 65 years). In total, 656 hospitalized patients aged ≥ 18 years (24.2% sarcopenic) were included in the study. The costs of hospitalization for patients aged < 65 years increased by EUR 1240 (95% confidence interval (CI): EUR 596–1887), while increased costs of EUR 721 (95% CI: EUR 13–1429) were observed for those aged ≥ 65 years [156]. An increase in disability-related hospitalizations and nursing home placement, as well as higher household healthcare expenses, is associated with increased health care costs. Since sarcopenia increases disability costs, sarcopenia is expected to lead to high public health costs. At present, there are very few economic data available on sarcopenia. Economic data on sarcopenia are currently very limited. There has only ever been one study on the healthcare costs related to sarcopenia in the United States [5]. As a result of these estimates, the direct costs of sarcopenia reached USD 18.5 billion in 2000, USD 10.8 billion for men, and USD 7.7 billion for women. As a result, hospitalization, nursing home admissions, and home healthcare expenditures contribute to these costs. Approximately 1.5% of total health spending in the United States was devoted to this area in 2000. Additionally, sarcopenia is associated with multiple comorbid conditions, such as osteoporosis, obesity, and type II diabetes. Sarcopenia may bring even greater economic burden than reported in the Janssen study when these comorbidities and costs of healthcare are considered. There has yet to be a comprehensive economic assessment of sarcopenia in Europe, and the Janssen study is the first of its kind. Despite the lack of other economic assessments, many studies have been conducted regarding the relationships between sarcopenia and the costs of hospitalization and nursing home admission. One study conducted in the United Kingdom showed that sarcopenic patients had significantly longer hospital stays than patients without sarcopenia (mean of 13.4  ±  8.8 days for sarcopenic subjects versus 9.4  ± 7.0 days for non-sarcopenic subjects; *p* = 0.003). Sarcopenia is associated with a loss of productivity, a decline in quality of life, and a loss of autonomy. Sarcopenia’s indirect costs, however, have not been assessed, either in the United States or in Europe. Janssen et al. [5] investigated how pharmacological treatment, public health campaigns, and physical activity interventions could reduce the prevalence of sarcopenia in the United States, as well as its effect on healthcare spending overall. According to their findings, a 10% reduction in sarcopenia prevalence would result in a USD 1.1 billion (dollars adjusted to 2000 rate) annual saving in the US. It is important to consider this potential economic savings in public health contexts. It is startling to find that there are no public health campaigns for reducing the prevalence of sarcopenia, despite the economic costs of osteoporotic fractures being similar. Health policy decision-makers should consider economic investments in sarcopenia prevention and treatment to ensure significant savings in the future, especially in light of the growing number of older people worldwide [25].

## 5. Conclusions

Even though we encountered differences between studies from different regions of the world regarding the methods used to measure muscle mass and to estimate sarcopenia parameters, we found that sarcopenia is a common disorder among elderly people regardless of sex, ethnicity, or region. The onset of sarcopenia is still related to aging; a timely diagnosis may help to prevent some negative health effects. Identifying the pathogenesis of sarcopenia is the main objective of a modern approach to understanding this intriguing condition. As sarcopenia is a multifactorial condition, it is crucial to emphasize the relevance of the diverse risk factors that contribute to it. In this review, we summarized 13 relevant factors related to this pathogenesis that should be considered before designing any research related to sarcopenia. We suggest that for developing a uniform consensus for screening of this disease we need to focus on the biology of sarcopenia. We found 50 biochemical markers from six pathways that have already been explored in subjects with sarcopenia. Therefore, we suggest that these summarized biomarkers should be evaluated further for future diagnosis to help determine the biology of this disease, thereby contributing to further investigations. This may also help to establish a uniform consensus for screening and defining this disease. Associated with a series of adverse economic and social implications such as disability, hospitalization, and death, sarcopenia is a disease of the elderly characterized by the loss of both muscle mass and strength; as a result, the condition is associated with an increased cost of medical care. We suggest an urgent need for the development of strategies, including exercise, to delay sarcopenia prevalence by utilizing smart technologies in the COVID-19 era.

## Data Availability

Not applicable.

## Abbreviations

EWGSOP	European Working Group on Sarcopenia for Older People
IWGA	International Working Group on Sarcopenia
AWGS	Asian Working Group for Sarcopenia
FINH	Foundation for the National Institutes of Health
BIA	Bioimpedance analysis
SARC-F	Strength, assistance with walking, rising from a chair, climbing stairs, and falls
HRQoL	Health-related quality of life
SarQoL	Sarcopenia and Quality of Life
COPD	Chronic obstructive pulmonary disease
PUD	Peptic ulcer disease
KNHANES	Korean National Health and Nutrition Examination Survey
SNPs	Single nucleotide polymorphisms
GWAS	Genome-wide association scan
TRHR	Thyrotropin-releasing hormone receptor
LBM	Lean body mass
ACE	Angiotensin I-converting enzyme I
MSTN	Myostatin
ACTN3	Alpha actinin 3
CNTF	Ciliary neurotrophic factor
VDR	Vitamin D receptor
IGF1	Insulin-like growth factor 1
IL-6	Interleukin-6
CAV1	Caveolin-1
FTO	Fat mass and obesity-associated
LMI	Lean mass index
NUDT3	Nudix hydrolase 3
KLF5	Kruppel-like Factor 5
HLA-DQB1-AS1	HLA-DQB1 antisense RNA 1
MTHFR	Methylenetetrahydrofolate reductase
ACTN3	Alpha-actinin-3
NRF2	Nuclear respiratory factor 2
HLA-DRB1	HLA class II histocompatibility antigen-DRB1 beta chain
GDF5	Growth differentiation factor 5
DYM	Dymeclin
DLEU1	Deleted in lymphocytic leukemia 1
SLC39A8	Solute carrier family 39 member 8
RN7SKP297	RN7SK pseudogene 297
C12orf60	Chromosome 12 open reading frame 60
RBBP6	Retinoblastoma-binding protein 6
ALDH1A2	Aldehyde dehydrogenase 1 family member A2
TGF α	Transforming growth factor alpha
ZBTB38	Zinc finger and BTB domain-containing protein 38
BRSK1	BR serine/threonine kinase 1
AOC1	Amine oxidase copper-containing 1
ZNF678	Zinc finger protein 678
GEO	Gene Expression Omnibus
P3NP	Procollagen type III N-terminal peptide
HtrA1	High temperature requirement serine protease A1
PF&S	Physical frailty and sarcopenia
MNA	Mini Nutritional Assessment
PS	Possible sarcopenia
MM	Muscle mechanography
RCAC	Residential care apartment complex
CAF	C-terminal agrin fragments
NMJs	Neuromuscular junction
SPPB	Short Physical Performance Battery
DHEA	Dehydroepiandrosterone
TGF	Transforming growth factors
FST	Follistatin
GDF-15	Growth differentiation factor 15
BMPs	Bone morphogenic proteins
IR	Irisin
BDNF	Brain-derived neurotrophic factor
FABP3	Fatty acid-binding protein 3
P3NP	N-terminal peptide
3MH	3-methylhistidine
C1q	Complement component 1q
CRP	C-reactive protein
TNF	Tumor necrosis factor
IL	Interleukin
HSP72	Heat shock 70 kDa protein 1
MIP-1β	Macrophage inflammatory protein 1β
MIF	Macrophage migration inhibitory factor
b-CHE	Butyryl-cholinesterase
oxLDL	Oxidized low-density lipoprotein
